# Crosstalk prohibition at the deep-subwavelength scale by epsilon-near-zero claddings

**DOI:** 10.1515/nanoph-2023-0085

**Published:** 2023-04-25

**Authors:** Wenjie Ji, Jie Luo, Hongchen Chu, Xiaoxi Zhou, Xiangdong Meng, Ruwen Peng, Mu Wang, Yun Lai

**Affiliations:** National Laboratory of Solid State Microstructures, School of Physics, and Collaborative Innovation Center of Advanced Microstructures, Nanjing University, Nanjing 210093, China; School of Physical Science and Technology, Institute of Theoretical and Applied Physics, Soochow University, Suzhou 215006, China; School of Optical and Electronic Information, Suzhou City University, Suzhou 215000, China

**Keywords:** crosstalk prohibition, epsilon-near-zero media, evanescent waves, waveguides

## Abstract

To prevent the crosstalk between adjacent waveguides in photonic integrated circuits, the minimum thickness of the cladding layers is around half a wavelength, which imposes a fundamental limitation to further integration and miniaturization of photonic circuits. Here, we reveal that epsilon-near-zero claddings, either isotropic or anisotropic, can break the above bottleneck by prohibiting the crosstalk for the modes with magnetic field polarized in the *z* direction at a deep-subwavelength thickness (e.g., *λ*_0_/30, *λ*_0_ is the free-space wavelength), therefore bestowing ultra-compact waveguide systems. The physical origin of this remarkable effect attributes to the divergent impedance of epsilon-near-zero materials far beyond those of dielectric or epsilon-negative claddings. Through full-wave simulations and microwave experiments, we have verified the effectiveness of the ultrathin epsilon-near-zero cladding in crosstalk prohibition. Our finding reveals the significant impact of impedance difference in waveguide designs and opens a promising route toward ultra-compact photonic chips.

## 1 Introduction

Ultra-compact and high-performance photonic integrated circuits have been identified as a key enabling technology that provides a promising solution to the current bottleneck in conventional micro-electronics [1, 2]. Similar to their electronic counterparts, photonic integrated circuits face the fundamental challenge of further integration and miniaturization. In order to prevent the crosstalk between adjacent waveguides, the cladding layer between them is usually required to have a minimum thickness of ∼*λ*_0_/2, where *λ*_0_ is free-space wavelength. This limitation imposes a fundamental barrier to further increasing the density of waveguides in photonic integrated circuits. Recently, by using innovative strategies such as anisotropic cladding [3], [4], [5], [6], [7], [8], [9], [10], [11], waveguide superlattices [12], [13], [14], and sinusoidally curved waveguides [15], [16], [17], the cladding thickness has been reduced to the level of ∼*λ*_0_/3. However, further reduction has met great difficulties. Very recently, by introducing photonic crystal waveguides with shifted dispersions, the cladding layers have been completely removed [18], but such an approach requires photonic crystals and thus significantly increases the difficulty of fabrication. To date, both academia and industry are still waiting a practical route to further reduce the thickness of cladding layers to the deep-subwavelength regime, which could enormously boost dense on-chip integration.

In this work, we propose that near-zero-index materials (NZIMs) [19], [20], [21], [22], [23], [24], [25], [26], [27], [28], [29], [30], [31], [32], [33], [34], [35], [36], [37], and more specifically, epsilon-near-zero (ENZ) media [23], [24], [25], [26] provide a solution to this fundamental bottleneck problem. The NZIMs represent a unique type of materials with near-zero permittivity or/and permeability. Such materials are practical and have been realized in various frequencies from microwaves to THz, infrared, and visible spectra [19], [20], [21], [22], [23], [24], [25], [26], [27], [28], [29], [30]. Based on NZIMs, a plethora of novel phenomena have been demonstrated, including tunneling waveguides [38], [39], [40], radiation and flux control [41], [42], [43], photonic doping and anti-doping [44], [45], [46], [47], [48], cloaking [49, 50], loss-induced field and absorption enhancement [51], [52], [53], nonlinearity enhancement [24, 54], [55], [56], and zero Minkowski-canonical momentum [57], etc. However, so far, there has been little attention on exploiting such materials as the cladding layers in photonic waveguide systems.

Here, we point out that ENZ claddings exhibit a significant advantage over all previous types of claddings such as dielectric/air and epsilon-negative (ENG) materials, i.e., a substantially large impedance for the polarization with magnetic field along the *z* direction, which diverges as *ɛ* → 0. This remarkable property bestows ENZ claddings the possibility of breaking the limitation of minimum cladding thickness in photonic waveguides, thus leading to much more compact waveguide systems for the modes with magnetic field polarized in the *z* direction. Through an analysis based on the coupled-mode theory, we find that ENZ claddings, either isotropic or anisotropic, can reduce the crosstalk between two adjacent waveguides to nearly zero with a thickness of deep-subwavelength scale, e.g., ∼*λ*_0_/30. Through full-wave simulations and microwave experiments based on a waveguide-type ENZ cladding, we have demonstrated the prohibition of crosstalk with such an ultrathin thickness — an exceptional capability beyond both dielectric/air and ENG claddings. Further for practical applications, an ultra-compact waveguide array is designed and demonstrated numerically using ENZ claddings of *λ*_0_/30 at the optical communication wavelength of *λ*_0_ = 1550 nm. This finding results from exploiting the large impedance contrast induced by ENZ materials in waveguide systems, which opens a gate toward photonic integrated circuits with extreme compactness.

## 2 Results

### 2.1 The critical role of impedance

The crosstalk between adjacent waveguides originates from the evanescent waves in the cladding. Traditionally, in order to guarantee negligible crosstalk, the cladding thickness is preferred to be large enough such that the amplitude of evanescent waves decays to a negligible level across the thickness. Usually, this can only be achieved by a thickness larger than *λ*_0_/2. Recent studies suggest that increasing the anisotropy of the claddings could further enhance the decay rate of the evanescent waves and thus efficiently suppress the crosstalk [3], [4], [5], [6], [7], [8], [9], [10], [11]. Here, we reveal that besides the decay rate, the impedance of claddings also plays an important role suppressing crosstalk. The impedance contrast between the core and cladding determines the amplitude of the evanescent waves on the interface between them. Since the ENZ cladding has a substantial impedance for the polarization with magnetic field along the *z* direction, the amplitude of the evanescent waves is significantly reduced on the interface between the ENZ cladding and the core. In this way, although the decay rate inside the ENZ cladding is not extremely large, the amplitude of evanescent waves can still be reduced enormously. When the relative permittivity of the ENZ cladding approaches zero, the impedance approaches infinity for all modes with magnetic field polarized in the *z* direction, and the mission of crosstalk prohibition can be accomplished by a near-zero thickness, which is in the deep-subwavelength scale. Our finding shows that the impedance contrast between the core and cladding has a significant impact on the required thickness of cladding for crosstalk prohibition, besides the decay rate that has been exploited extensively.

**Figure 1:**
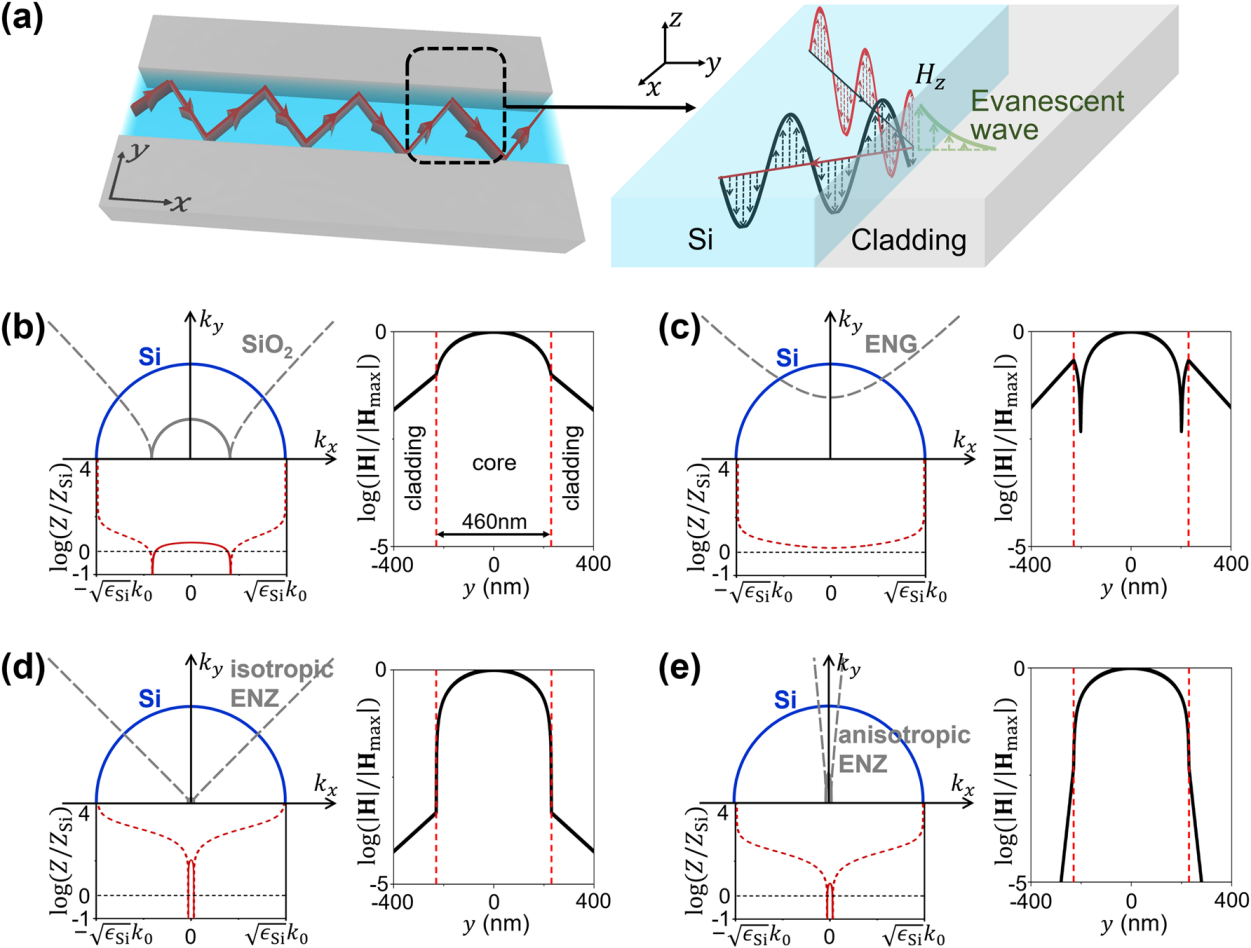
Physical properties of the ENZ claddings. (a) Schematic diagrams of a Si waveguide (left) and the mechanism of total reflection (right). The left panel graphs in (b)–(e) show the dispersion curves (top) and the impedance contrast (bottom) of four types of claddings, i.e., (b) SiO_2_, (c) ENG medium, (d) isotropic ENZ medium, and (e) anisotropic ENZ medium. The solid and dashed lines denote the real and imaginary parts of *k*_*y*_ (top, grey) and log(*Z*/*Z*_Si_) (bottom, red) as functions of *k*_*x*_. The blue lines correspond to the dispersion curve of Si. The right panel graphs in (b)–(e) show the distributions of normalized magnetic fields $\left|H\right|/|H_{\text{max}}|$ inside the waveguide consisting of a 460 nm-width core of Si and four types of claddings for the fundamental mode with magnetic field polarized in the *z* direction. **H**_max_ is the maximal magnetic field inside the waveguide.

To clearly illustrate the importance of impedance in the claddings, we consider the physical model of a waveguide with a transmission core of silicon (Si) with relative permittivity *ɛ*_Si_ = 12 sandwiched by the claddings of nonmagnetic media with relative permittivity tensor $\left(\begin{array}{cc} \varepsilon_{x} & 0\\ 0 & \varepsilon_{y} \end{array}\right)$ at the wavelength of *λ*_0_ = 1550 nm, as illustrated in Figure 1a. The right panel graph shows the origin of total reflection, where the evanescent waves are excited in the claddings, leading to the undesired crosstalk. Now, we consider the polarization with magnetic field along the *z* direction. The dispersion relation and wave impedance (i.e., the ratio of the tangential electric and magnetic fields) are expressed as $k_{y}^{2}/\varepsilon_{x}+k_{x}^{2}/\varepsilon_{y}=k_{0}^{2}$ and $Z=\sqrt{k_{0}^{2}-k_{x}^{2}/\varepsilon_{y}}/\left(\omega \varepsilon_{0}\sqrt{\varepsilon_{x}}\right)$ [58], respectively. Here *k*_0_ is the wave number in free space. *k*_*x*_ and *k*_*y*_ are the *x*- and *y*-components of wave vector, respectively. *ω* is the angular frequency, and *ɛ*_0_ is the permittivity of free space. The left panel graphs in Figure 1b–e show, respectively, the dispersion curves (top) and impedance contrast *Z*/*Z*_Si_ (bottom) of four types of claddings, i.e. dielectric (SiO_2_, $\varepsilon_{x}=\varepsilon_{y}=\varepsilon_{\text{Si}O_{2}}=2.07$ ), ENG media (*ɛ*_*x*_ = *ɛ*_*y*_ = *ɛ*_ENG_ = −5), isotropic ENZ media (*ɛ*_*x*_ = *ɛ*_*y*_ = 0.01) and anisotropic ENZ media (*ɛ*_*x*_ = 1, *ɛ*_*y*_ = 0.01). Here, *Z*_Si_ ( $=\sqrt{\varepsilon_{\text{Si}}k_{0}^{2}-k_{x}^{2}}/\left(\omega \varepsilon_{0}\varepsilon_{\text{Si}}\right)$ ) is the wave impedance of Si. The solid and dashed lines denote, respectively, the real and imaginary parts of *k*_*y*_ (top, grey) or *Z*/*Z*_Si_ (bottom, red) as functions of *k*_*x*_. The blue lines correspond to the dispersion curve of Si, which is a circle with a radius of $\sqrt{\varepsilon_{\text{Si}}}k_{0}$ .

In the case of SiO_2_ cladding (Figure 1b), the dispersion curve is a hyperbola in the regime of $\sqrt{\varepsilon_{\text{Si}O_{2}}}k_{0}<|k_{x}|\leq \sqrt{\varepsilon_{\text{Si}}}k_{0}$ , where both *k*_*y*_ and *Z* are imaginary numbers. The imaginary *k*_*y*_ is the signature of the total reflection as well as the formation of evanescent waves inside the cladding [1, 2], the magnitude of which determines how fast the evanescent waves decay inside the cladding. When the cladding is composed of ENG media (Figure 1c), the dispersion curve is a hyperbola of a different orientation, and the *k*_*y*_ is imaginary for any *k*_*x*_ because the ENG media cannot support any propagating wave. In the cases of the isotropic ENZ (Figure 1d) and anisotropic ENZ (Figure 1e) claddings, the dispersion curves become linear lines, which is reasonable as the parameter of *ɛ* = 0 or *ɛ*_*y*_ = 0 represents a critical transition point between the dielectric and ENG cases. It is seen that the *k*_*y*_ is imaginary for any *k*_*x*_ ≠ 0, indicating that total reflection occurs except for normal incidence. This property infers that ENZ media can serve as the claddings of waveguides with an excellent tolerance on the propagation constants of the waveguide modes, i.e., *β* = *k*_*x*_ ≠ 0. Interestingly, the imaginary *k*_*y*_ in Figure 1e diverges rapidly with increasing *k*_*x*_. This means that the anisotropic ENZ claddings could have an extreme decay rate. This is induced by the extreme anisotropy (i.e., *ɛ*_*x*_ ≫*ɛ*_*y*_) and the details of derivation are presented in Supporting Information.

However, the decay rate is not the whole story of how good claddings work. Besides the decay rate, the impedance contrast between the cladding and core is another crucial factor that determines the effectiveness of claddings, but was rarely discussed in the literature. Unlike the decay rate that determines how fast the evanescent waves decay in the cladding, the impedance contrast determines the amplitude of the evanescent waves on the interface between the cladding and core. In Figure 1b–e, it is seen that the impedance contrast *Z*/*Z*_Si_ of both isotropic and anisotropic ENZ claddings is several orders higher than that of the SiO_2_ and ENG claddings. Such a colossal impedance contrast for the polarization with magnetic field along the *z* direction is a unique result of near-zero *ɛ* or *ɛ*_*y*_ [59] which diverges when *ɛ* or *ɛ*_*y*_ approaches zero. Due to the large impedance contrast, despite the fact that the decay rate of isotropic ENZ cladding is comparable to that of the SiO_2_ and ENG claddings, the effectiveness of isotropic ENZ cladding is much better. For anisotropic ENZ cladding, the combination of significant decay rate and large impedance contrast makes a super cladding.

The right panel graphs in Figure 1b–e show the distributions of normalized magnetic fields inside a waveguide consisting of a Si core with 460 nm width and four types of claddings (i.e., SiO_2_, ENG, isotropic ENZ, and anisotropic ENZ media). The fundamental mode with magnetic field polarized in the *z* direction is excited. We can clearly see that the magnetic fields are stable on the Si-SiO_2_ and Si-ENG interfaces, but drop abruptly on the Si-isotropic ENZ and Si-anisotropic ENZ interfaces. This is a fundamental difference between ENZ claddings and all other types of claddings. Due to this physical mechanism, the ENZ claddings in principle can eliminate the crosstalk with an ultra-thin thickness of deep-subwavelength scale, which is an impossible for the traditional dielectric/air or ENG claddings.

The functionality of the significant impedance contrast can be easily understood from the reflection coefficient (regarding to the magnetic field) at the Si-ENZ interface, which is r = (*Z*_Si_ − *Z*)/(*Z*_Si_ + *Z*). When *Z* ≫ *Z*_Si_, we have r = −1. This implies that the incident and reflected waves have the same amplitude, but a π phase difference, thus leading to a near-zero total magnetic field at the interface. Consequently, the magnetic field inside the ENZ claddings must be near-zero, as implied by the boundary conditions, and therefore the evanescent waves inside the ENZ claddings almost disappear.

### 2.2 Analysis of waveguide coupling

**Figure 2:**
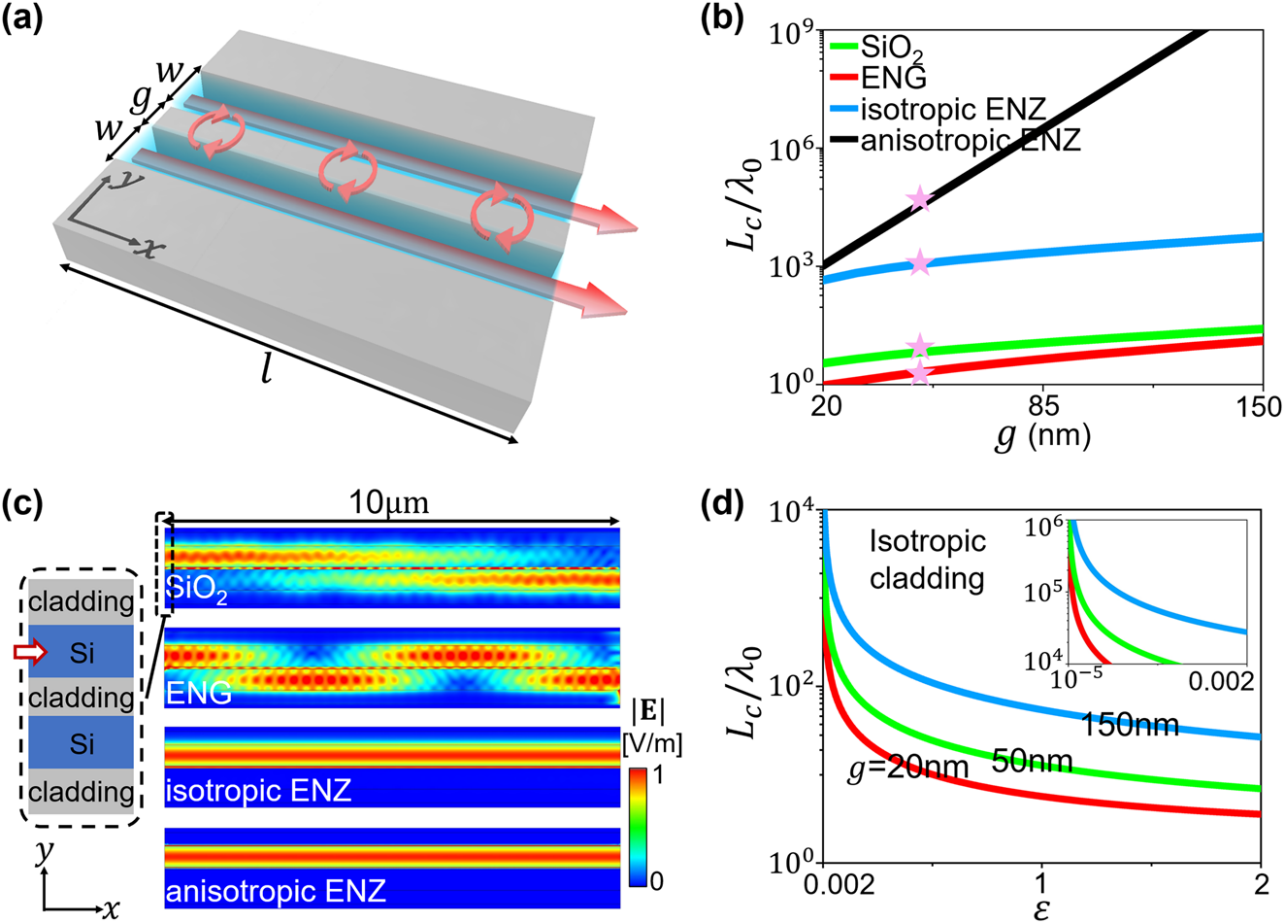
Prohibition of crosstalk between two Si waveguides. (a) Schematic layout of two Si waveguides with width *w*, separation distance *g*, and length *l*. (b) Normalized coupling length *L*_c_/*λ*_0_ of the fundamental mode with magnetic field polarized in the *z* direction in the case of *w* = 460 nm for four types of claddings with different *g*. (c) Distributions of electric-field amplitude $\left|E\right|$ for the four types of claddings. The fundamental mode of the upper Si waveguide is excited on the left port, as illustrated by the inset. Here, we set *w* = 460 nm, *g* = 50 nm (∼*λ*_0_/30, marked by stars in (b)) and *l* = 10 μm. (d) *L*_c_/*λ*_0_ as a function of the relative permittivity *ɛ* of isotropic cladding for *g* = 20 nm (red), 50 nm (green), and 150 nm (blue). The inset shows the range of 10^−5^ ≤ *ɛ* ≤ 0.002.

We perform theoretical analysis and numerical simulations to confirm the remarkable functionality of the ENZ claddings. For simplicity, we consider two Si waveguides with the same width *w* and length *l* separated by a separation distance of *g*, as schematically shown in Figure 2a. The operating wavelength is chosen as *λ*_0_ = 1550 nm. Figure 2b shows the normalized coupling length *L*_c_/*λ*_0_ of the fundamental mode with magnetic field polarized in the *z* direction in the case of *w* = 460 nm for the SiO_2_, ENG, isotropic ENZ, and anisotropic ENZ claddings with different *g*. The coupling length *L*_c_ is defined such that the power would transfer completely from the first waveguide to the adjacent waveguide after a propagating distance of *L*_c_. Here, *L*_c_ is calculated based on the coupled-mode theory. According to the coupled-mode theory, the mth-order mode dispersion of the coupled waveguides with a nonmagnetic cladding of $\left(\begin{array}{cc} \varepsilon_{x} & 0\\ 0 & \varepsilon_{y} \end{array}\right)$ can be derived as (see Supporting Information),

(1) $$\left\{\begin{array}{c} kw=\arctan\left(\frac{\varepsilon_{\text{Si}}}{\varepsilon_{x}}\frac{\alpha }{k}\right)+\arctan\left[\frac{\varepsilon_{\text{Si}}}{\varepsilon_{x}}\frac{\alpha }{k}\tanh\left(\frac{\alpha g}{2}\right)\right]+m\pi \;\\ kw=\arctan\left(\frac{\varepsilon_{\text{Si}}}{\varepsilon_{x}}\frac{\alpha }{k}\right)+\arctan\left[\frac{\varepsilon_{\text{Si}}}{\varepsilon_{x}}\frac{\alpha }{k}\coth\left(\frac{\alpha g}{2}\right)\right]+m\pi \; \end{array}\right.$$

for symmetric and antisymmetric modes, respectively. Here $k=\sqrt{\varepsilon_{\text{Si}}k_{0}^{2}-\beta_{s\left(a\right)}^{2}}$ and $\alpha =\sqrt{\varepsilon_{x}\left(\frac{\beta_{s\left(a\right)}^{2}}{\varepsilon_{y}}-k_{0}^{2}\right)}$ , where *β*_*s*_ and *β*_*a*_ denote the propagation constants of symmetric and antisymmetric modes, respectively. *m* (= 0, 1, 2, …) refers to the order of waveguide modes.

Then, the coupling length *L*_*c*_ between the two waveguides can be evaluated as [60].

(2) $$L_{c}=\frac{\pi }{\left|\beta_{s}-\beta_{a}\right|}.$$

Based on Equations (1) and (2), we calculate and plot the *L*_c_ for different types of claddings in Figure 2b. It is clearly seen that the *L*_c_ of the ENZ claddings is several orders larger than that of the SiO_2_ and ENG cladding, implying that the crosstalk between the two Si waveguides is significantly reduced by the ENZ claddings. This is because under the limit of *ɛ*_*y*_ → 0, we have

(3) $$\beta_{s}=\beta_{a}.$$

Equation (3) indicates that *L*_*c*_ → ∞, irrespective of *g*, which is also valid for the isotropic case of *ɛ*_*x*_ = *ɛ*_*y*_ = *ɛ* → 0. It is noteworthy that this conclusion is valid for any waveguide mode with magnetic field polarized in the *z* direction as the equality *β*_*s*_ = *β*_*a*_ is *m*-independent (see numerical proofs in Supporting Information).

Then, we perform full-wave finite-element simulations using the commercial software COMSOL Multiphysics to verify the prohibition of crosstalk, as shown in Figure 2c. Here, the width is *w* = 460 nm, the separation distance is *g* = 50 nm, and the length is *l* = 10 μm. We note that *g* ∼ *λ*_0_/30, indicating deep-subwavelength claddings. The fundamental mode with magnetic field polarized in the *z* direction in the upper waveguide is excited on the left input port, as illustrated by the inset. Figure 2c shows the distributions of the electric-field amplitude for the four types of claddings. Clearly, strong coupling appears between the two Si waveguides for the SiO_2_ or ENG claddings. The coupling lengths are observed to be ∼10.7μm for the SiO_2_ cladding and ∼3.2μm for the ENG cladding, which agree well with the theoretical prediction in Figure 2b ( ∼10.5μm and ∼3.3μm ). Remarkably, for the cases of isotropic ENZ and anisotropic ENZ claddings, the coupling almost completely disappears and there is no wave or signal in the adjacent waveguide. The theoretical coupling length is 1.8 × 10^3^μm for the isotropic ENZ cladding and 6.546 × 10^4^μm for the anisotropic ENZ cladding, far beyond that of the SiO_2_ and ENG claddings. We should emphasize that when *ɛ* (isotropic case) or *ɛ*_*y*_ (anisotropic case) of claddings approaches zero, the coupling length goes to infinity, i.e., *L*_*c*_ → ∞. For verification, we plot the coupling length *L*_c_ for an isotropic cladding with different thickness *g* in Figure 2d, which confirms that *L*_c_ → ∞ when *ɛ* → 0, no matter how small *g* is.

### 2.3 Microwave experiment

**Figure 3:**
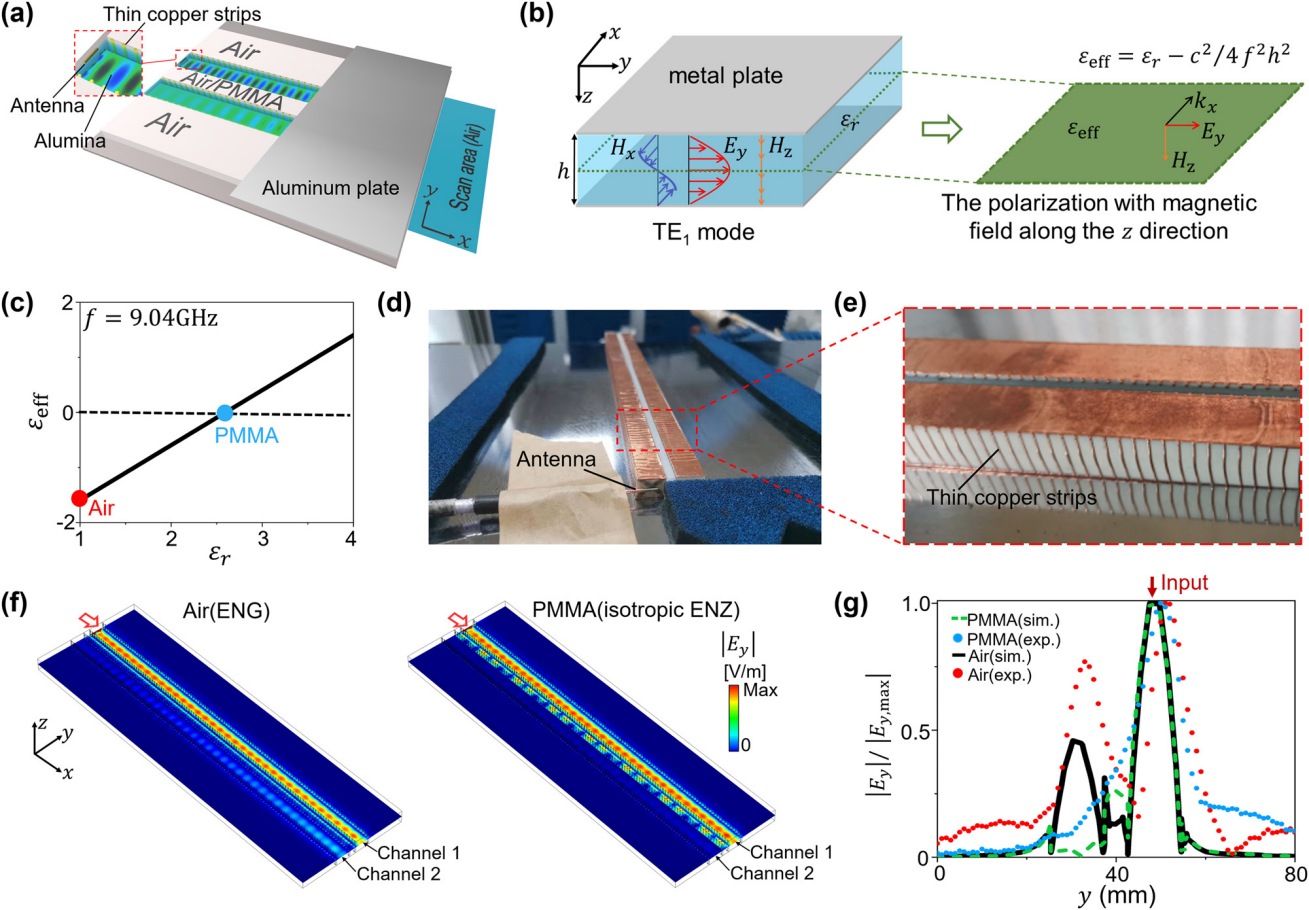
Experimental verification. (a) Schematic of the experimental setup of a PPW. Inside the PPW, two alumina bars are placed along the *x* direction working as two dielectric waveguides. Periodic thin copper strips are vertically wrapped around the alumina bars to eliminate the fundamental TEM mode. A horizontally oriented antenna is placed nearby the left port of the upper alumina bar to excite the TE_1_ mode. (b) Illustrations of TE_1_ mode in a PPW (left) and an effective two-dimensional medium for the polarization with magnetic field along the *z* direction (right). (c) Effective relative permittivity *ɛ*_eff_ as a function of *ɛ*_*r*_ of the filling material inside the PPW at 9.04 GHz. (d) Photograph of the experimental sample and antenna. (e) Zoomed-in photograph of the thin copper strips. (f) Simulated distributions of |*E*_*y*_| on the middle plane of the PPW for air (effectively ENG, left) and PMMA (effectively isotropic ENZ, right) claddings. (g) Experimental measured (lines) and simulated (dots) normalized |*E*_*y*_| to its maximal value |*E*_*y*,max_| on the exiting surface for PMMA (effectively isotropic ENZ) and air (effectively ENG) claddings with *g* = 5.2 mm.

In the following, we experimentally demonstrate the prohibition of crosstalk by using an isotropic ENZ cladding with deep-subwavelength thickness in the microwave regime. Here, the low-loss isotropic ENZ cladding is realized by waveguide metamaterials based on parallel-plate waveguides (PPWs) [26, 36, 44, 61], [62], [63], [64], [65]. Figure 3a shows the schematic of the experimental setup. Two parallel aluminum plates of the PPW are separated vertically by a distance of *h* = 10.3 mm. Now, we consider the TE_1_ mode in this PPW, which is dominated by *E*_*y*_, *H*_*x*_, and *H*_*z*_, as illustrated in the left panel of Figure 3b. On the middle plane of the PPW, there are only non-zero *E*_*y*_ and *H*_*z*_. Under this circumstance, the TE_1_ mode-dominated PPW can be viewed as a two-dimensional effective medium where the polarization of magnetic field is along the *z* direction (right panel of Figure 3b). In this way, we can effectively regard the experimental PPW model with TE_1_ mode shown in Figure 3a as the aforementioned waveguide system where the magnetic field is polarized in the *z* direction. The effective relative permittivity of the waveguide metamaterial is [26, 36, 44, 61], [62], [63], [64], [65].

(4) $$\varepsilon_{\text{eff}}=\varepsilon_{r}-c^{2}/4f^{2}h^{2},$$

where *f* is the operating frequency, *c* is the speed of light in vacuum, and *ɛ*_*r*_ is the relative permittivity of the filling dielectric inside the PPW. From Equation (4), we see that for fixed *h* and *f*, the *ɛ*_eff_ can be adjusted to be negative, zero, and positive values through engineering the *ɛ*_*r*_.

To implement the waveguide system with isotropic ENZ claddings, we utilize two 280 mm-long alumina (*ɛ*_*r*_ = 9.2) bars along the *x* direction with the same width of 12 mm and a separation distance of *g* = 5.2 mm in the *y* direction. They work as two coupled dielectric waveguides. Thin copper strips of area 0.05 mm^2^ and a pitch of 2.5 mm are placed vertically around the alumina strips to eliminate the fundamental TEM mode (with electric field along the *z* direction) of the PPW [36, 44, 61], [62], [63], [64], [65], as shown in Figure 3e. The working frequency is chosen to be *f* = 9.04 GHz. According to Equation (4), we find that the *ɛ*_eff_ of this waveguide metamaterial filled with alumina (air) are 6.6 (−1.6). This indicates that this waveguide metamaterial filled with air can effectively serve as an ENG medium, which has been proposed to realize low-loss effective surface plasmon polaritons [61, 62]. Then, we insert a PMMA bar (*ɛ*_*r*_ = 2.6) between the two alumina bars. The *ɛ*_eff_ of the PMMA-filled waveguide metamaterial turns out to be near-zero (Figure 3c), thus it can effectively serve as an isotropic ENZ cladding.

In the microwave experiment, we use a horizontally oriented antenna to excite the TE_1_ mode of the PPW from the left port of the upper alumina bar and measure the electric field *E*_*y*_ on the exiting surface of area 20 mm × 80 mm, as illustrated in Figure 3a. The measurement is performed using a PNA Network Analyzer N5224B. The photos of experimental samples and antenna are presented in Figure 3d. In the experiments, in order to avoid the situation that the mode has returned back in the first channel at the end of waveguide, the length of the fabricated waveguide is chosen to be shorter than the coupling length, and at the same time, long enough to show the difference between the models with air (effectively ENG) and PMMA (effectively isotropic ENZ) claddings. Figure 3f shows the simulated distributions of |*E*_*y*_| for air (left) and PMMA (right) claddings with *g* = 5.2 mm on the middle plane of the PPW. We can see a clear difference of field distributions at the end of the waveguides with air and PMMA claddings, which confirms the crosstalk suppression by the PMMA cladding. Figure 3g displays the experimentally measured normalized |*E*_*y*_| along the exiting surface along the *y* direction (dots), which agrees well with the simulation results (lines), for both PMMA (effectively isotropic ENZ) and air (effectively ENG) claddings. In the case of ENG cladding, two obvious peaks appear where the two alumina waveguides are located, indicating evident crosstalk between the two waveguides. While in the case of isotropic ENZ cladding there is only one peak located in the input waveguide, indicating that the crosstalk is low. These experimental and simulation results have verified the functionality of crosstalk prohibition by the ultra-thin isotropic ENZ cladding (∼*λ*_0_/6).

We note that the mismatch between experimental and simulation results could be caused by two main factors. First, it is hard to excite the modes in the channel 1 only, while completely preventing the excitation of modes in the adjacent channel 2 on the waveguide port, because the two channels are very close to each other. Second, the imperfection of the alumina strips may cause scattered waves that would couple into the adjacent waveguide.

### 2.4 Ultra-compact waveguide arrays

**Figure 4:**
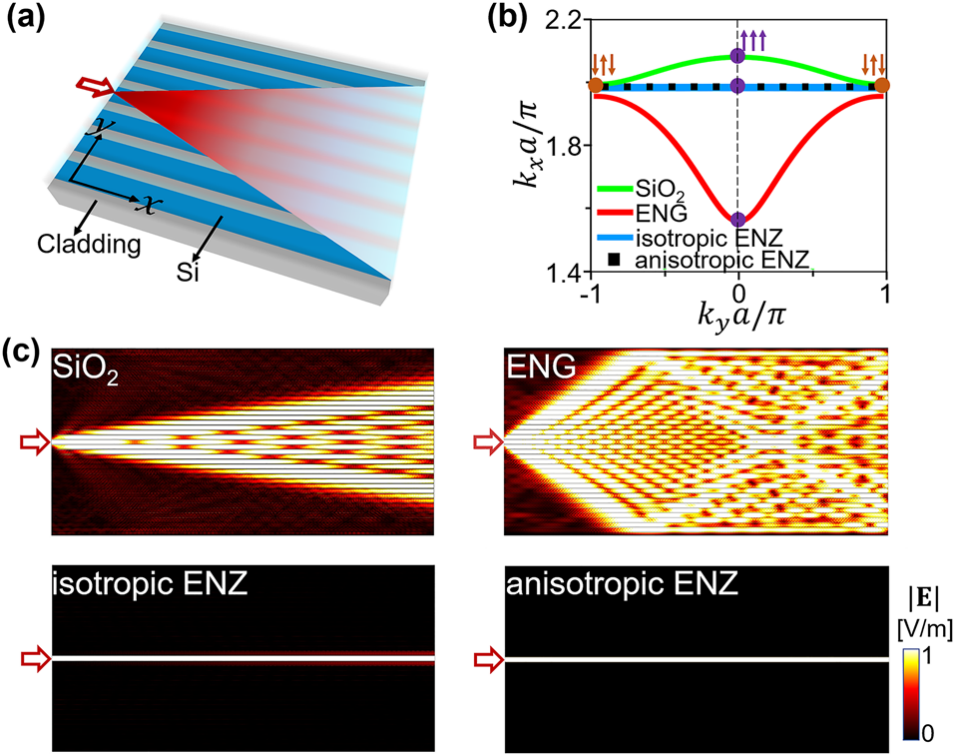
Ultra-compact waveguide arrays. (a) Schematic diagram of a waveguide array. (b) Dispersion relation of the waveguide array with SiO_2_ (green), ENG (red), isotropic ENZ (blue) and anisotropic ENZ (black) claddings at *λ*_0_ = 1550 nm. The purple (brown) marks denote the symmetric (antisymmetric) mode at *k*_*y*_ *a* = 0 (*k*_*y*_ *a* = ±*π*). (c) Distributions of $\left|E\right|$ in the waveguide arrays with the SiO_2_, ENG, isotropic ENZ, and anisotropic ENZ claddings. The fundamental waveguide mode with magnetic field polarized in the *z* direction is excited in the central waveguide.

We utilize the above crosstalk prohibition based on ENZ claddings to realize ultra-compact waveguide arrays. Figure 4a illustrates the schematic of an array of Si waveguides. Each waveguide has a width *w*, and their separation distance (edge-to-edge) is *g*. Based on the coupled-mode theory, the dispersion relation of the waveguide array with a pitch of *a* (= *w* + *g*) is expressed as [17, 66].

(5) $$k_{x}=\beta +2C\cos\left(k_{y}a\right),$$

where *β* is the propagation constant for an isolated waveguide, and *C* = (*β*_*s*_ − *β*_*a*_)/2 is the coupling coefficient between two waveguides. Here *β*_*s*_ (or *β*_*a*_) is the propagation constant of the symmetric (antisymmetric) mode in the waveguide array, which equals to *k*_*x*_ when *k*_*y*_ *a* = 0 (or *k*_*y*_ *a* = ±π), as marked by the arrows in Figure 4b. Equation (5) indicates that a flat dispersion curve, i.e., *k*_*x*_ = *β*, appears in the absence of coupling (i.e., *C* = 0), which is a criterion of zero crosstalk in a waveguide array.

Figure 4b presents the exact dispersion relation of the Si waveguide array at *λ*_0_ = 1550 nm calculated by using the transfer matrix method [67]. Here, we set the widths of the Si waveguide and cladding as *w* = 460 nm and *g* = 50 nm, respectively. Four types of claddings, i.e., SiO_2_, ENG, isotropic ENZ, and anisotropic ENZ claddings with the same parameters in Figure 1, are applied here. It is clearly seen that *k*_*x*_ is independent of *k*_*y*_ for the isotropic ENZ and anisotropic ENZ claddings, indicating that the crosstalk is nearly zero in the waveguide array with such deep-subwavelength (∼*λ*_0_/30) ENZ claddings. On the contrary, *k*_*x*_ varies with *k*_*y*_ for the SiO_2_ or ENG claddings, indicating the existence of coupling between waveguides. For further verification, in Figure 4c, we show the simulated distributions of electric-field amplitude in a 40 μm-long waveguide array by exciting the fundamental mode with magnetic field polarized in the *z* direction in the central waveguide. The simulation results clearly show that the wave propagating in the central waveguide couples to the adjacent waveguides and spreads out for the SiO_2_ or ENG claddings, but remains confined in the central waveguide for the isotropic ENZ and anisotropic ENZ claddings. Therefore, the crosstalk in the waveguide array is reduced to zero with the ENZ claddings.

### 2.5 On-chip strip waveguides

**Figure 5:**
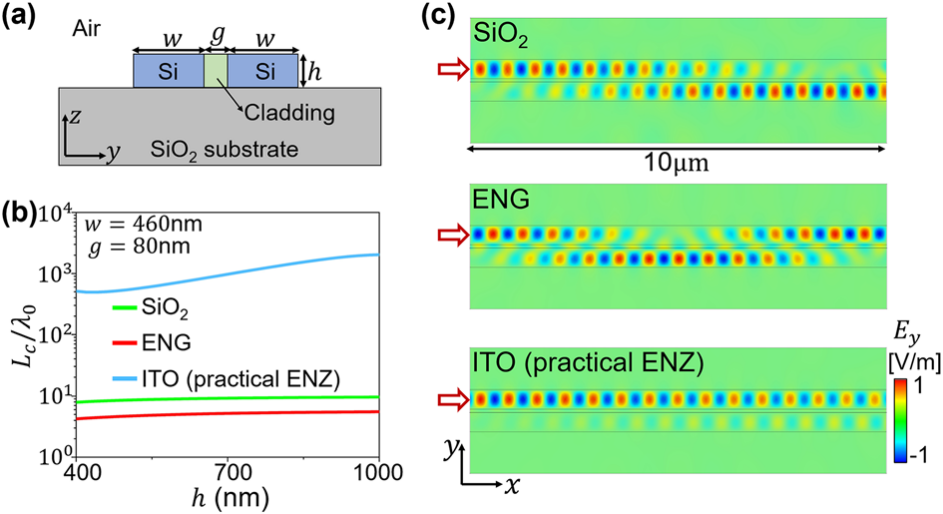
On-chip strip waveguide. (a) Schematic layout of the cross-section of two coupled on-chip Si waveguides with width *w*, separation distance *g* and height *h*. (b) Normalized coupling length *L*_c_/*λ*_0_ as a function of *h* for SiO_2_, ENG, and ITO (practical ENZ) claddings in the case of *w* = 460 nm, *g* = 80 nm. (c) Distributions of *E*_*y*_ in two coupled Si strip waveguides with SiO_2_, ENG, and ITO claddings at *λ*_0_ = 1550 nm. The fundamental TE mode is excited in the upper Si waveguide on the left port. The relevant parameters are *w* = 460 nm, *g* = 80 nm, and *h* = 220 nm.

In the following, we would like to show a practical implementation of on-chip Si strip waveguides using a practical ENZ medium, i.e., indium tin oxide (ITO) claddings. A 80 nm-thick ITO film is characterized by a relative permittivity around 0.01 + 0.45*i* at *λ*_0_ = 1550 nm [68], which can approximately serve as an isotropic ENZ medium. Figure 5a illustrates the cross-section of the Si strip waveguide consisting of two Si strips (width *w* = 460 nm, height *h*) separated by a cladding layer (separation distance *g* = 80 nm, height *h*). Based on Equation (2), we calculate the normalized coupling length *L*_*c*_/*λ*_0_ in this waveguide through evaluating *β*_*s*_ and *β*_*a*_ in simulations. In Figure 5b, we see that the coupling length *L*_*c*_/*λ*_0_ of the fundamental TE mode in the waveguide with ITO claddings is much larger than those in the waveguides with SiO_2_ or ENG claddings, and it is almost irrespective of the waveguide height *h*, indicating excellent performance of crosstalk suppression by the ITO claddings. Figure 5c shows the top view of simulated *E*_*y*_-distribution on the *xy* plane for claddings of SiO_2_ (upper), ENG (middle), and ITO (lower), respectively. Clearly, at the deep-subwavelength thickness of *g* ∼ *λ*_0_/20, evident crosstalk appears for SiO_2_ and ENG claddings, while the crosstalk is largely suppressed by the ITO claddings.

## 3 Discussion and conclusions

Unlike the previous works that focus on increasing the decay rate in the claddings [3], [4], [5], [6], [7], [8], [9], [10], [11], here we utilize impedance as a tool to achieve the extraordinary ability of crosstalk suppression with an ultrathin cladding in the deep-subwavelength scale. The impedance contrast between the cladding and core determines the amplitude of evanescent waves on their interfaces, which is another crucial factor that determines the effectiveness of the cladding besides the decay rate. The principle of crosstalk suppression based on impedance contrast is universal and applies to general waveguides such as bending waveguides (see examples in Supporting Information) and all waveguide modes. We note that in bending waveguides the ENZ claddings can prevent the leakage of waveguide modes irrespective of bending angles. Here, our demonstration is based on modes with magnetic field polarized in the *z* direction in a slab waveguide system because we have only considered the ENZ claddings. If the cladding is made of mu-near-zero materials with near-zero effective permeability, then the impedance is divergent for all modes with electric field polarized in the *z* direction. Naturally, with epsilon-mu-near-zero claddings whose effective permittivity and permeability are both near-zero, the impedance will be divergent for any waveguide mode (see details in Supporting Information).

Practical implementation of the ENZ medium in the near-infrared can be realized by conductive materials like cadmium oxide (CdO) [52, 69, 70], doped semiconductors [71], ITO [54, 68, 72], aluminum- or gallium-doped zinc oxide [29, 73], erbium ion-doped aluminum oxide (Er^3+^:Al_2_O_3_) [28], etc. Some materials, such as ITO and Er^3+^:Al_2_O_3_, can be conveniently combined with Si or other dielectric waveguides [28, 72]. The permittivity of a practical ENZ medium has a small but finite value due to material losses. In Supporting Information, we have considered the influence of the loss and we find that the crosstalk can still be drastically reduced by lossy ENZ claddings, exhibiting a much longer coupling length than waveguides with SiO_2_ or ENG claddings of the same thickness. Currently, the main barrier of applying ENZ media into waveguide systems is loss, but this in principle could be relieved in the future by using practical photonic crystal designs as NZIMs [30], [31], [32], [33], [34] or adding gain into the system to compensate the loss [74], [75], [76].

The bandwidth issue is also important for communications. The bandwidth of the effective isotropic ENZ media based on the microwave metallic waveguide demonstrated in Figure 3 is about 350 MHz (8.86 GHz–9.21 GHz), which is large enough for microwave wireless communication in which a typical transmission channel of information occupies around 60 MHz. For the case of optical communication, the ENZ region of the ITO for $\left|\varepsilon_{\text{ITO}}\right|<0.2$ in Figure 5c is from 1520 to 1580 nm [68]. This indicates that the functionality of the ENZ claddings can cover a substantial spectral range for practical use.

## Supporting Information

The online version of this article offers Supplementary Material.

## Supplementary Material

Supplementary Material Details
