# Supplementary material for: Crosstalk prohibition at the deep-subwavelength scale by epsilon-near-zero claddings
Source: Nanophotonics. 2023 Apr 25;12(11):2007–17. doi: 10.1515/nanoph-2023-0085 (PMC11502104; doi:10.1515/nanoph-2023-0085)
Supplement: Supplementary file 1 — Supplementary Material Details [file j_nanoph-2023-0085_suppl_001.pdf]

Supporting Information

Crosstalk prohibition at the deep-subwavelength scale by epsilon-near-zero claddings

Wenjie Ji, Jie Luo, Hongchen Chu, Xiaoxi Zhou, Xiangdong Meng, Ruwen Peng,* Mu Wang,* and Yun Lai**

W. Ji, H. Chu, X. Meng, R. Peng, M. Wang, Y. Lai

National Laboratory of Solid State Microstructures, School of Physics, and Collaborative Innovation Center of Advanced Microstructures, Nanjing University, Nanjing 210093, China.

E-mail: rwpeng@nju.edu.cn; muwang@nju.edu.cn; laiyun@nju.edu.cn

J. Luo, X. Zhou

Institute of Theoretical and Applied Physics, School of Physical Science and Technology, Soochow University, Suzhou 215006, China

E-mail: luojie@suda.edu.cn

1. **Dispersion relation and coupling length of two coupled slab waveguides**
2. **Suppression of crosstalk for high-order waveguide modes**
3. **Suppression of crosstalk in bending waveguides**
4. **Mu-near-zero (MNZ) and epsilon-and-mu-near-zero (EMNZ) claddings**
5. **Loss effect of ITO claddings**
6. **Suppression of crosstalk by non-ideal ENZ claddings**

1. Dispersion relation and coupling length of two coupled slab waveguides

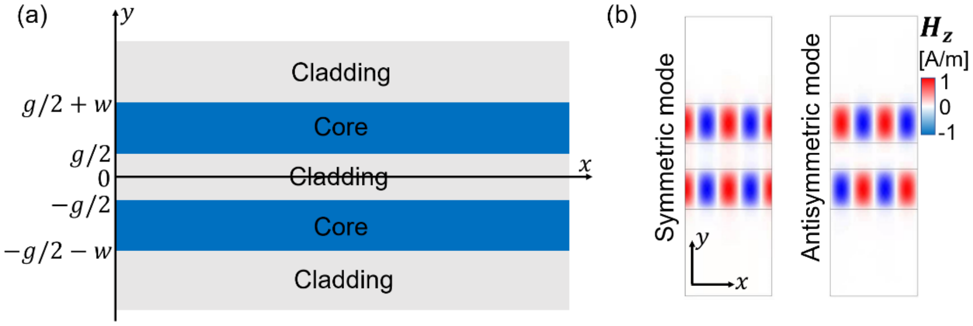

Figure S1. (a) Illustration of two coupled slab waveguides. (b) Distributions of H_z for the fundamental symmetric (left) and antisymmetric (right) modes with magnetic field polarized in the z direction at $\lambda_0 = 1550\text{nm}$. The waveguide system consists of Si cores and SiO_2 claddings with $w = 460\text{nm}$ and $g = 300\text{nm}$.

We consider two coupled slab waveguides with a width of w of each waveguide and an edge-to-edge separation of g, as schematically shown in Figure S1(a). We assume the waveguide core (or cladding) is characterized by a relative permittivity tensor $\begin{pmatrix} \varepsilon_{x,\text{co}} & & \\ & \varepsilon_{y,\text{co}} & \\ & & \varepsilon_{z,\text{co}} \end{pmatrix}$ (or $\begin{pmatrix} \varepsilon_{x,\text{cl}} & & \\ & \varepsilon_{y,\text{cl}} & \\ & & \varepsilon_{z,\text{cl}} \end{pmatrix}$) and a relative permeability tensor $\begin{pmatrix} \mu_{x,\text{co}} & & \\ & \mu_{y,\text{co}} & \\ & & \mu_{z,\text{co}} \end{pmatrix}$ (or $\begin{pmatrix} \mu_{x,\text{cl}} & & \\ & \mu_{y,\text{cl}} & \\ & & \mu_{z,\text{cl}} \end{pmatrix}$). The field $\phi(y)$, i.e. H_z (E_z) for the mode with magnetic field polarized in the z direction (the mode with electric field polarized in the z direction), in different regions can be written as,

$$\phi(y) = \begin{cases} A_1 e^{-\alpha_{\text{cl}}\left(y-\frac{g}{2}-w\right)} & \left(y > \frac{g}{2} + w\right) \\ A_2 \cos\left[k_{\text{co}}\left(y - \frac{g}{2}\right) + \varphi\right] & \left(\frac{g}{2} + w \geq y \geq \frac{g}{2}\right) \\ A_3\left[e^{\alpha_{\text{cl}}\left(y-\frac{g}{2}\right)} \pm e^{-\alpha_{\text{cl}}\left(y+\frac{g}{2}\right)}\right] & \left(\frac{g}{2} > y \geq 0\right), \end{cases} \tag{S1}$$

where $k_{\text{co}} = \sqrt{(k_0^2\mu_{z,\text{co}} - \beta^2/\varepsilon_{y,\text{co}})\varepsilon_{x,\text{co}}}$, $\alpha_{\text{cl}} = \sqrt{\varepsilon_{x,\text{cl}}(\frac{\beta^2}{\varepsilon_{y,\text{cl}}} - k_0^2\mu_{z,\text{cl}})}$ for the mode with magnetic field polarized in the z direction, and $k_{\text{co}} = \sqrt{(k_0^2\varepsilon_{z,\text{co}} - \beta^2/\mu_{y,\text{co}})\mu_{x,\text{co}}}$, $\alpha_{\text{cl}} = \sqrt{\mu_{x,\text{cl}}(\frac{\beta^2}{\mu_{y,\text{cl}}} - k_0^2\varepsilon_{z,\text{cl}})}$ for the mode with electric field polarized in the z direction. k_0 is the wave number in free space, and β is the

propagation constant. A_1, A_2 and A_3 are amplitudes of fields. The sign $+(-)$ denotes the symmetric (antisymmetric) eigenmode.

Here, we note that the $|\alpha_{\mathrm{cl}}|$ denotes the decay rate of evanescent waves in claddings. When $\varepsilon_{x,\mathrm{cl}} \gg \varepsilon_{y,\mathrm{cl}}$, we have $|\alpha_{\mathrm{cl}}| \gg \beta$ for non-zero β. As a consequence, for anisotropic ENZ claddings with $\varepsilon_{x,\mathrm{cl}} \gg \varepsilon_{y,\mathrm{cl}} \to 0$, we would have an extremely large decay rate in claddings and an extraordinarily long coupling length in coupled waveguide systems, as observed in Figure 1 and 2 in the main text.

Imposing the boundary conditions that $\phi(y)$ must be continuous on the interfaces, the $\phi(y)$ can be rewritten as,

$$\phi(y) = \begin{cases} A\cos(k_{\mathrm{co}}w + \varphi)e^{-\alpha_{\mathrm{cl}}(y-\frac{g}{2}-w)} & (y > \frac{g}{2} + w) \\ A\cos\left[k_{\mathrm{co}}\left(y - \frac{g}{2}\right) + \varphi\right] & \left(\frac{g}{2} + w \geq y \geq \frac{g}{2}\right) \\ \frac{A\cos\varphi(e^{\alpha_{\mathrm{cl}}y} \pm e^{-\alpha_{\mathrm{cl}}y})}{e^{\frac{\alpha_{\mathrm{cl}}g}{2}} \pm e^{-\frac{\alpha_{\mathrm{cl}}g}{2}}} & (g/2 > y \geq 0) \end{cases} \tag{S2}$$

with $A = A_2$. Then, considering the boundary condition that E_x must be also continuous on the interfaces, we obtain the dispersion relations of symmetric and antisymmetric modes with magnetic field polarized in the z direction of the coupled-waveguide system as,

$$kw = \arctan\left(\frac{\varepsilon_{x,\mathrm{co}}}{\varepsilon_{x,\mathrm{cl}}}\frac{\alpha}{k}\right) + \arctan\left[\frac{\varepsilon_{x,\mathrm{co}}}{\varepsilon_{x,\mathrm{cl}}}\frac{\alpha}{k}\tanh\left(\frac{\alpha g}{2}\right)\right] + m\pi \tag{S3}$$

for symmetric modes, and

$$kw = \arctan\left(\frac{\varepsilon_{x,\mathrm{co}}}{\varepsilon_{x,\mathrm{cl}}}\frac{\alpha}{k}\right) + \arctan\left[\frac{\varepsilon_{x,\mathrm{co}}}{\varepsilon_{x,\mathrm{cl}}}\frac{\alpha}{k}\coth\left(\frac{\alpha g}{2}\right)\right] + m\pi \tag{S4}$$

for antisymmetric modes. Here $k = \sqrt{(k_0^2\mu_{z,\mathrm{co}} - \beta_{s(a)}^2/\varepsilon_{y,\mathrm{co}})\varepsilon_{x,\mathrm{co}}}$ and $\alpha = \sqrt{\varepsilon_{x,\mathrm{cl}}(\frac{\beta_{s(a)}^2}{\varepsilon_{y,\mathrm{cl}}} - k_0^2\mu_{z,\mathrm{cl}})}$, where β_s and β_a denote propagation constants of symmetric and antisymmetric modes, respectively. m $(= 0,1,2,\ldots)$ refers to the order of waveguide modes. For illustration, Figure S1(b) presents the distributions of H_z of the fundamental symmetric (left) and antisymmetric (right) modes in two coupled waveguides composed of Si cores and SiO_2 claddings with $w = 460\mathrm{nm}$ and $g = 300\mathrm{nm}$ at $\lambda_0 = 1550\mathrm{nm}$.

Similarly, for the mode with electric field polarized in the z direction, considering the continuity of H_x on the interfaces, we obtain the dispersion relations of symmetric and antisymmetric modes as,

$$kw = \arctan\left(\frac{\mu_{x,\mathrm{co}}}{\mu_{x,\mathrm{cl}}}\frac{\alpha}{k}\right) + \arctan\left[\frac{\mu_{x,\mathrm{co}}}{\mu_{x,\mathrm{cl}}}\frac{\alpha}{k}\tanh\left(\frac{\alpha g}{2}\right)\right] + m\pi \tag{S5}$$

for symmetric modes, and

$$kw = \arctan\left(\frac{\mu_{x,\mathrm{co}}}{\mu_{x,\mathrm{cl}}}\frac{\alpha}{k}\right) + \arctan\left[\frac{\mu_{x,\mathrm{co}}}{\mu_{x,\mathrm{cl}}}\frac{\alpha}{k}\coth\left(\frac{\alpha g}{2}\right)\right] + m\pi \tag{S6}$$

for antisymmetric modes. Here, $k = \sqrt{(k_0^2\varepsilon_{z,\mathrm{co}} - \beta_{s(a)}^2/\mu_{y,\mathrm{co}})\mu_{x,\mathrm{co}}}$, and $\alpha = \sqrt{\mu_{x,\mathrm{cl}}(\frac{\beta_{s(a)}^2}{\mu_{y,\mathrm{cl}}} - k_0^2\varepsilon_{z,\mathrm{cl}})}$.

Then, based on the β_s and β_a from Equation S3-S4 (or Equation S5-S6) for the modes with magnetic field polarized in the z direction (or the mode with electric field polarized in the z direction), the coupling length L_c between the two waveguides can be evaluated as (see e.g. *J. Opt. Soc. Am. A 11, 963 (1994)*)

$$L_c = \frac{\pi}{|\beta_s - \beta_a|}. \tag{S7}$$

2. Suppression of crosstalk for high-order waveguide modes

In the main text, near-complete suppression of waveguide crosstalk by using ENZ claddings is demonstrated for the fundamental mode with magnetic field polarized in the z direction. Here, Figure S2 demonstrates that for high-order modes, the waveguide crosstalk can also be suppressed by isotropic ENZ or anisotropic ENZ claddings. For comparison, SiO_2 and ENG claddings are compared here, showing strong waveguide crosstalk.

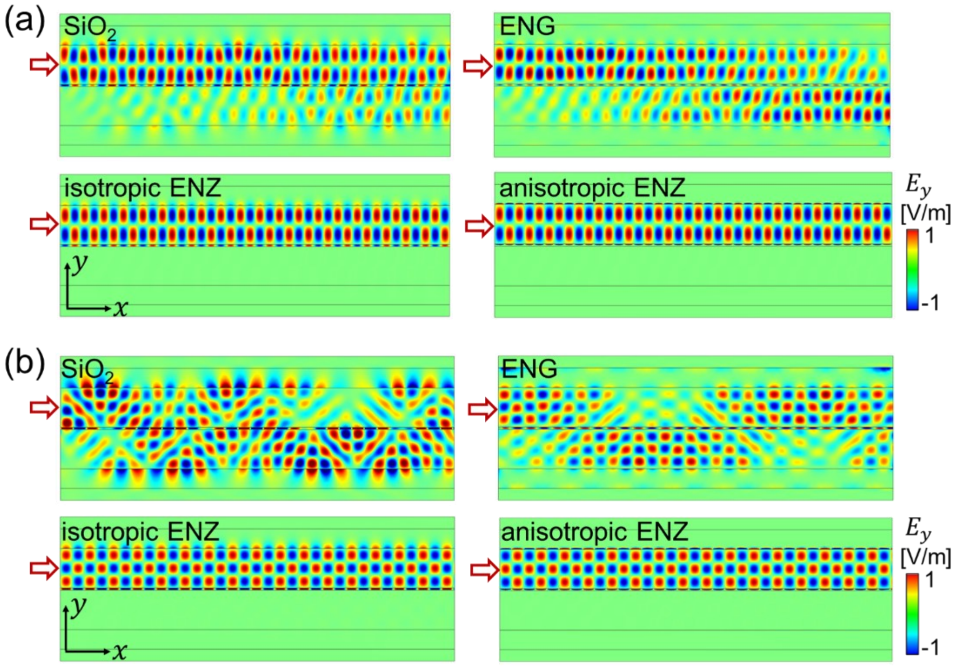

Figure S2. Distributions of E_y of different orders of the modes with magnetic field polarized in the z direction modes in a coupled-waveguide system composed of Si cores and four different types of claddings (i.e., SiO_2, ENG, isotropic ENZ, anisotropic ENZ) with $w = 1000\text{nm}$ and $g = 50\text{nm}$ at $\lambda_0 = 1550\text{nm}$.

3. Suppression of crosstalk in bending waveguides

In the main text, near-zero crosstalk in straight slab waveguides with ENZ claddings is demonstrated. Here, we show that the ENZ claddings can also be exploited to significantly suppress crosstalk in bending waveguides. Figure S3(a) shows that the crosstalk is negligibly weak in a $180°$ waveguide bend consisting of two coupled Si waveguides with isotropic ENZ claddings. For comparison, we successively utilize SiO_2, ENG, isotropic ENZ and anisotropic ENZ media as the claddings and calculate the transmission (port 1 to port 2) with respect to the bending radius r, as presented in Figure S3(b). We see that transmission is low for SiO_2 or ENG claddings. Interestingly, the transmission for the deep-subwavelength isotropic ENZ and anisotropic ENZ claddings is near-100%, irrespective of the bending radius r.

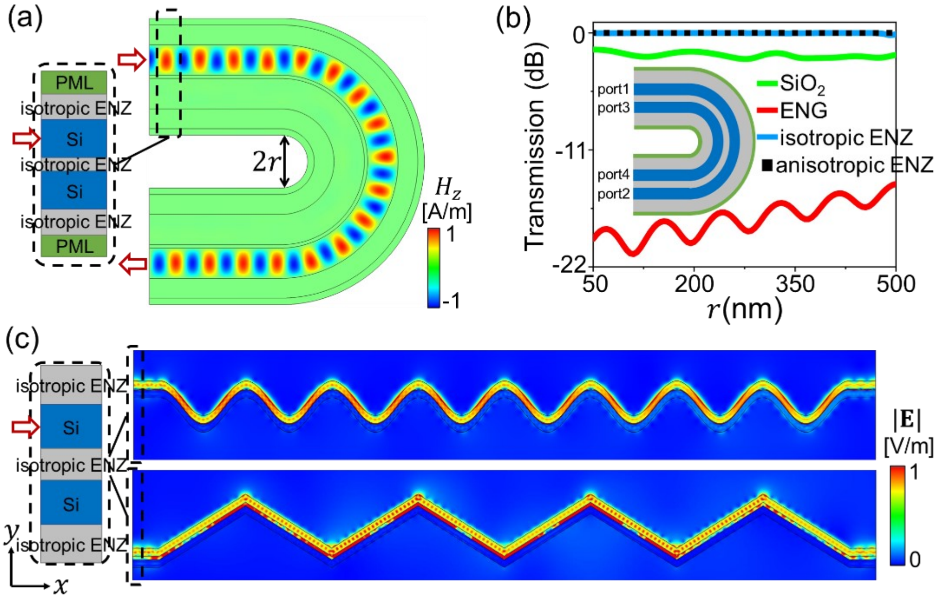

Figure S3. (a) Distributions of H_z in a bending waveguide ($w = 460$nm, $g = 50$nm, $r = 400$nm) with isotropic ENZ claddings. (b) Transmission (port 1 to port 2) in the bending waveguide with SiO_2, ENG, isotropic ENZ or anisotropic ENZ claddings. (c) Distributions of electric-field amplitude in 43.1μm-long sinusoidally curved (upper) and Z-shape (lower) waveguides with isotropic ENZ claddings at $\lambda_0 = 1550$nm. We set $w = 460$nm and $g = 50$nm. The light signal is sent to the left port of the upper Si waveguide.

Furthermore, Figure S3(c) shows the simulated electric-field amplitude in 43.1μm-long sinusoidally curved (upper) and Z-shaped (lower) waveguides with isotropic ENZ claddings at $\lambda_0 = 1550$nm. The light signal is sent to the upper Si waveguide (see the inset). We find that the transmission efficiency is ~0.91 (or ~0.96) in the sinusoidally curved (or Z-shaped) waveguide, demonstrating the extraordinary ability of crosstalk suppression of the ENZ media in bending waveguides.

4. Mu-near-zero (MNZ) and epsilon-and-mu-near-zero (EMNZ) claddings

In the main text, we find that ENZ claddings can be used to realize near-zero waveguide crosstalk for the mode with magnetic field polarized in the z direction. Here, we show that mu-near-zero (MNZ) claddings can eliminate waveguide crosstalk for the mode

with electric field polarized in the z direction, and epsilon-and-mu-near-zero (EMNZ) media can eliminate waveguide crosstalk for both the two kinds of modes.

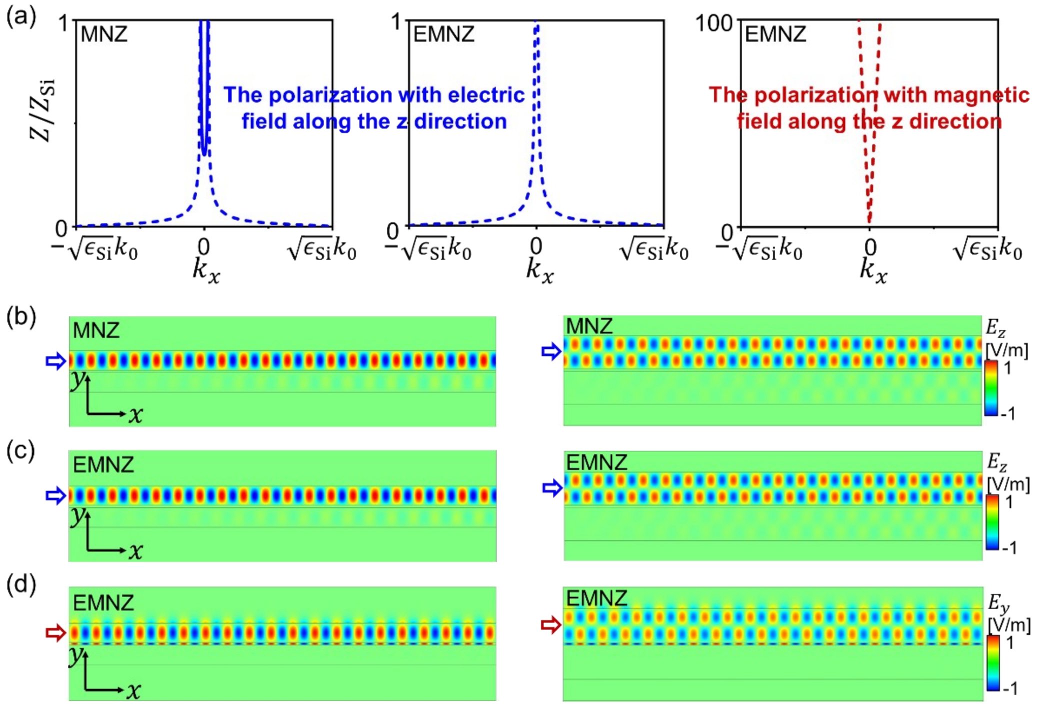

Figure S4. (a) Wave impedance contrast between Si and MNZ medium ($\varepsilon_r = 1,\ \mu_r = 0.01$, left) for the polarization with electric field along the z direction, Si and EMNZ medium ($\varepsilon_r = \mu_r = 0.01$) for the polarization with electric field along the z direction (middle) and the polarization with magnetic field along the z direction (right). The solid and dashes lines denote, respectively, the real and imaginary parts of impedance contrast as the function of k_x. [(b)-(d)] Distributions of electric field for the fundamental (left, $w = 460\text{nm},\ g = 50\text{nm}$) and 1st-order (right, $w = 800\text{nm},\ g = 50\text{nm}$) waveguide modes in two coupled Si slab waveguides with (b) MNZ claddings for the modes with electric field polarized in the z direction, (c) EMNZ claddings for the modes with electric field polarized in the z direction, (d) EMNZ claddings for the mode with magnetic field polarized in the z direction. The light signal of $\lambda_0 = 1550\text{nm}$ is sent to the left port of the upper Si waveguide.

First, we analyze the wave impedance of the MNZ and EMNZ media. Figure S4(a) presents the impedance contrast between Si and MNZ medium (relative permittivity

$\varepsilon_r = 1$, relative permeability $\mu_r = 0.01$) for the polarization with electric field along the z direction (left), EMNZ medium ($\varepsilon_r = \mu_r = 0.01$) for the polarization with electric field along the z direction (middle) and the polarization with magnetic field along the z direction (right). The solid and dashed lines denote, respectively, the real and imaginary parts of Z. We see that the wave impedance of the MNZ and EMNZ media for the polarization with electric field along the z direction becomes vanishingly small with increasing the k_x compared with that of Si, while the wave impedance of the EMNZ medium for the polarization with magnetic field along the z direction tends to be infinitely larger. Such an extreme impedance can prevent light penetrating into the MNZ and EMNZ media, thus suppressing the evanescent waves. Due to this extraordinary property, when the MNZ (EMNZ) medium is utilized as claddings in waveguides, the waveguide crosstalk for the modes with electric field polarized in the z direction (both the modes with electric field polarized in the z direction and the mode with magnetic field polarized in the z direction) can be eliminated.

For verification, Figure S4(b) shows the simulated electric-field distributions for the fundamental (left panel, $w = 460\text{nm}$, $g = 50\text{nm}$) and 1st-order (right panel, $w = 800\text{nm}$, $g = 50\text{nm}$) modes with electric field polarized in the z direction in two coupled Si slab waveguides when utilizing MNZ claddings. We see almost no waveguide crosstalk for the modes with electric field polarized in the z direction, irrespective of the order of waveguide modes. Figure S4(c) and S4(d) show the simulated electric-field distributions for the mode with electric field polarized in the z direction and the mode with magnetic field polarized in the z direction when utilizing EMNZ claddings, showing almost complete suppression of waveguide crosstalk for both the two kinds of modes, which is also independent of the order of waveguide modes.

5. Loss effect of ITO claddings

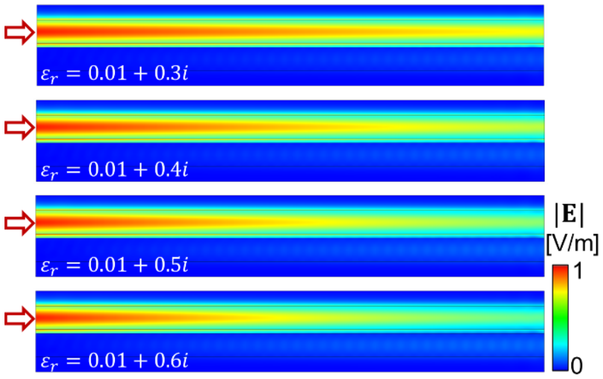

Figure S5. Distributions of electric-field amplitude in two 10μm-long coupled Si slab waveguides with ITO claddings of different loss tangent.

Here, we study the loss effect of indium tin oxide (ITO) claddings through investigating the coupling between two Si waveguides (with $w = 460\text{nm}$ and $g = 80\text{nm}$). Figure S5 shows the distributions of electric-field amplitude in two coupled Si slab waveguides when the ITO cladding possesses different imaginary part of permittivity (or loss tangent). Simulation results show that the crosstalk can still be reasonably suppressed. The similar behavior is also observed in two coupled Si strip waveguides (Figure S6). These results manifest that the waveguide crosstalk suppression using the ITO claddings is relatively robust against the material loss.

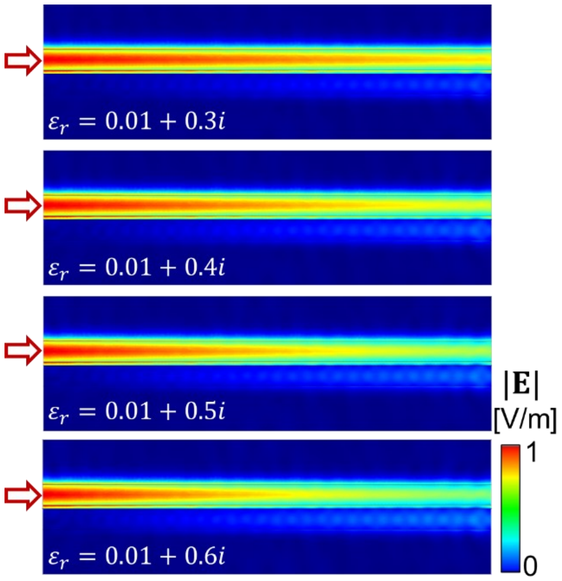

Figure S6. Distributions of electric-field amplitude in two 10μm-long coupled Si strip waveguides with ITO claddings of different loss tangent.

6. Suppression of crosstalk by non-ideal ENZ claddings

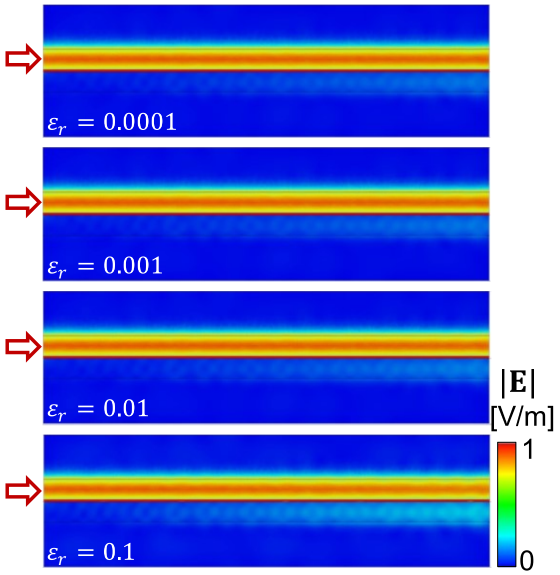

Figure S7. Distributions of electric-field amplitude in two 10μm-long coupled Si strip waveguides with isotropic ENZ claddings of different relative permittivities ε_r, where $w = 460\text{nm}$ and $g = 50\text{nm}$.

Figure S7 discusses the waveguide crosstalk suppression by non-ideal ENZ claddings, whose relative permittivity ε_r is not very close to zero. The simulation results show that excellent performance of crosstalk suppression can be obtained as long as $\varepsilon_r < 0.1$.
